# Is there a reduction in risk of revision when 36-mm heads instead of 32 mm are used in total hip arthroplasty for patients with proximal femur fractures? A matched analysis of 5,030 patients with a median of 2.5 years’ follow-up between 2006 and 2016 in the Nordic Arthroplasty Register Association

**DOI:** 10.1080/17453674.2020.1752559

**Published:** 2020-04-14

**Authors:** Georgios Tsikandylakis, Johan N Kärrholm, Geir Hallan, Ove Furnes, Antti Eskelinen, Keijo Mäkelä, Alma B Pedersen, Søren Overgaard, Maziar Mohaddes

**Affiliations:** a Department of Orthopaedics, Institute of Clinical Sciences, Sahlgrenska Academy, University of Gothenburg;; b The Swedish Hip Arthroplasty Register, Gothenburg, Sweden;; c Region Västra Götaland, Sahlgrenska University Hospital, Dept of Orthopaedics, Gothenburg, Sweden;; d The Norwegian Arthroplasty Register, Department of Orthopedic Surgery, Haukeland University Hospital, Norway;; e Department of Clinical Medicine, University of Bergen, Norway;; f Coxa Hospital of Joint Replacement, Tampere Finland;; g The Finnish Arthroplasty Register, Finland;; h Department of Orthopaedics and Traumatology, Turku University Hospital, Finland;; i Department of Clinical Epidemiology, Aarhus University Hospital, Denmark;; j The Danish Hip Arthroplasty Register, Denmark;; k Department of Orthopaedic Surgery and Traumatology, Odense University Hospital, Denmark;; l Institute of Clinical Research, University of Southern Denmark

## Abstract

Background and purpose — 32-mm heads are widely used in total hip arthroplasty (THA) in Scandinavia, while the proportion of 36-mm heads is increasing as they are expected to increase THA stability. We investigated whether the use of 36-mm heads in THA after proximal femur fracture (PFF) is associated with a lower risk of revision compared with 32-mm heads.

Patients and methods — We included 5,030 patients operated with THA due to PFF with 32- or 36-mm heads from the Nordic Arthroplasty Register Association database. Each patient with a 36-mm head was matched with a patient with a 32-mm head, using propensity score. The patients were operated between 2006 and 2016, with a metal or ceramic head on a polyethylene bearing. Cox proportional hazards models were fitted to estimate the unadjusted and adjusted hazard ratio (HR) with 95% confidence intervals (CI) for revision for any reason and revision due to dislocation for 36-mm heads compared with 32-mm heads.

Results — 36-mm heads had an HR of 0.9 (CI 0.7–1.2) for revision for any reason and 0.8 (CI 0.5–1.3) for revision due to dislocation compared with 32-mm heads at a median follow-up of 2.5 years (interquartile range 1–4.4).

Interpretation — We were not able to demonstrate any clinically relevant reduction of the risk of THA revision for any reason or due to dislocation when 36-mm heads were used versus 32-mm. Residual confounding due to lack of data on patient comorbidities and body mass index could bias our results.

During the past years total hip arthroplasty (THA) has become the preferred treatment option for displaced femoral neck fractures in even younger (55–64 years) patients (Rogmark et al. 2017). Previous studies have shown an increased risk of revision, especially due to dislocation, in patients receiving THA after proximal femur fracture (PFF) compared with patients operated due to primary osteoarthritis (OA) (Conroy et al. 2008, Hailer et al. 2012). The risk of THA dislocation in fracture patients varies widely from as low as 5% (Tabori-Jensen et al. 2019), especially when dual mobility cups (DMCs) are used, up to 6–18% (Burgers et al. 2012, Johansson 2014, Noticewala et al. 2018) with conventional cups. The risk of THA revision due to dislocation has been reported as even lower, ranging from 0.5 to 0.7% in national register studies (Conroy et al. 2008, Hailer et al. 2012), as not all unstable THAs are revised. According to the above-mentioned studies, increased age, male sex, the use of a posterior approach, and smaller head sizes are associated with increased risk of revision due to dislocation. To counteract the risk of dislocation, bigger head sizes have been used as they increase the impingement-free range of motion (Burroughs et al. 2005, Tsuda et al. 2016) and jumping distance of THA (Sariali et al. 2009). During the past years, the use of larger heads in THA has increased with 28-mm continuously declining and 32- and 36-mm increasing (Tsikandylakis et al. 2018b). However, register studies performed on patients with displaced femoral neck fracture (Jameson et al. 2012, Cebatorius et al. 2015) have not demonstrated any superiority of larger heads over smaller ones regarding risk of revision, especially due to dislocation. This effect has only been demonstrated in studies performed on a case mix of hip diagnoses that have reported an increased risk of revision due to dislocation when 28-mm or smaller heads are used compared with 32-mm or larger heads (Hailer et al. 2012, Kostensalo et al. 2013).

Most of the above-mentioned register studies have used 28-mm heads as reference, which are rarely used nowadays (Tsikandylakis et al. 2018b). Patients receiving THA after PFF have a higher risk for revision than patients with OA and should preferably be studied separately, setting 32 mm as contemporary standard of reference. We therefore investigated if increasing head size from 32 to 36 mm is associated with a decreased risk of revision, especially due to dislocation, in patients with PFF in the Nordic Arthroplasty Register Association (NARA) database. We hypothesized that the risk is lower when 36-mm heads are used.

## Patients and methods

This study was designed as a propensity-matched cohort study within NARA, a collaboration among the national arthroplasty registries of Denmark, Finland, Norway, and Sweden (Havelin et al. 2011).

**Figure 1.**
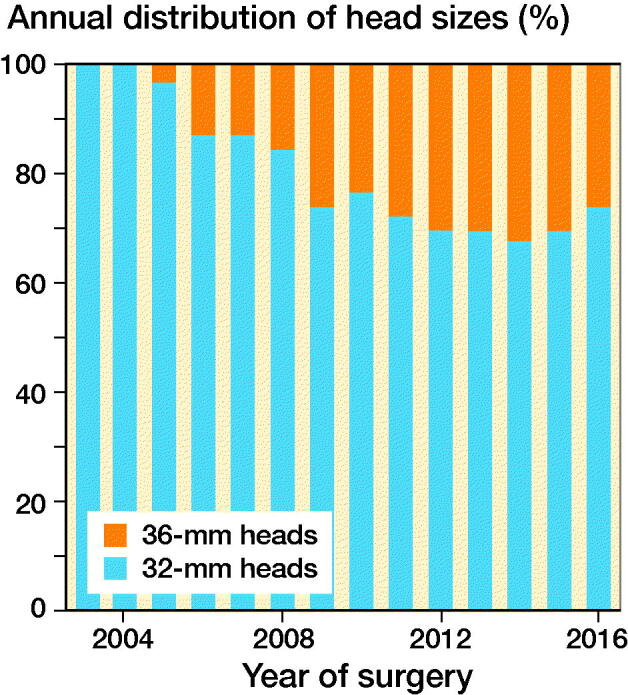
Use of 32- and 36-mm heads in MoXLPE and CoXLPE THA after proximal femur fracture in NARA database.

**Figure 2.**
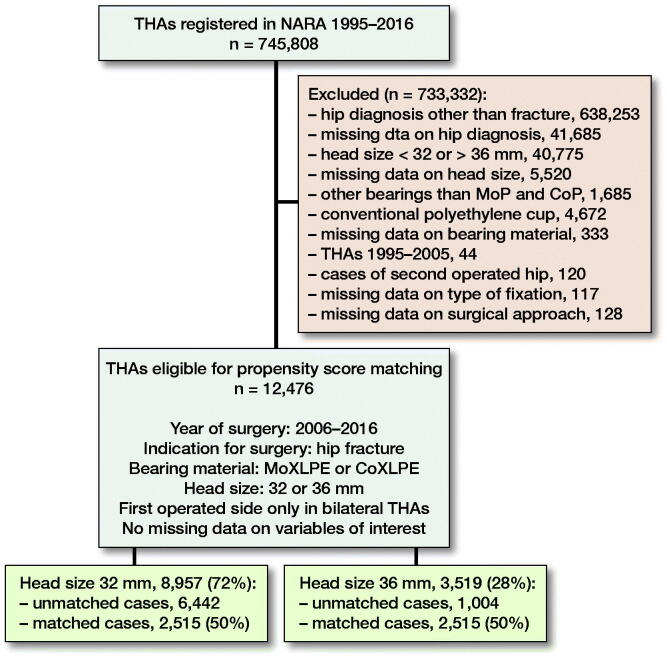
Flowchart of the selection and matching process.

We included patients operated with THA due to PFF, registered in the NARA database between January 1, 1995 and December 31, 2016. Patients operated with head sizes other than 32 or 36 mm, DMCs, and hip resurfacing were excluded. As metal on cross-linked polyethylene and ceramic on cross-linked polyethylene are the most common bearing types used in modern THA (Tsikandylakis et al. 2018b) we excluded all other bearing combinations. As 36-mm heads were not used in the Nordic countries until 2006 (Figure 1), we excluded all THAs performed before 2006. In patients with bilateral THA, the 2nd operated hip was excluded to fulfil the assumption of independent observations (Ranstam et al. 2011). The type of implant fixation included 4 categories: cemented, uncemented, hybrid, and reverse hybrid. The type of surgical approach is registered in NARA as either posterior or non-posterior because 1 of the national registries does not report further details on non-posterior approaches. Follow-up time, age, and year of surgery were handled as quantitative variables without grouping. Operations with any missing data on the above-mentioned variables were excluded (Figure 2).

After exclusions, 12,476 patients remained, of whom 72% had received a 32-mm and 28% a 36-mm head. There were differences between the groups as patients operated with 36-mm heads were younger with a higher proportion of males, operated more recently with predominantly a posterior approach and uncemented implant fixation (Table 1). These imbalances may confound the risk of THA revision, especially due to dislocation (Hailer et al. 2012, Jameson et al. 2012, Cebatorius et al. 2015). To reduce bias due to confounding, patients from the 36-mm group were matched to patients in the 32-mm group with a 1:1 ratio, using propensity score (PS) (Kuss et al. 2016) based on patient age, sex, year of surgery, type of implant fixation, bearing, and surgical approach. We were able to match 2,515 patients with 36-mm heads to 2,515 patients with 32-mm heads using the PS (Table 2). In the matched sample, the differences in sex, age, year of surgery, and type of surgical approach decreased considerably. We evaluated the balance of the covariates between the 2 head size groups before and after matching using absolute standardized differences in means (ASDM). The highest ASDM after matching was 0.1 (Table 2, Figure 3, see Supplementary data), below the threshold of 0.15 that indicates significant imbalance between groups (Austin 2011). Mortality rates were high in both head size groups (18–20%) but did not differ between them either before (Table 1) or after (Table 2) PS matching. In the matched sample median follow-up was 2.5 years (interquartile range 1–4.4).

**Figure 4.**
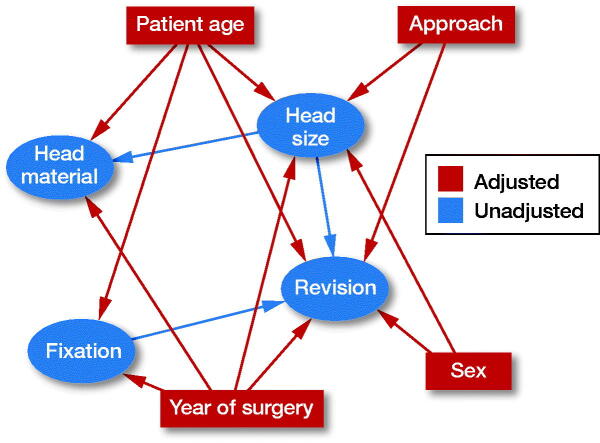
A directed acyclic graph (DAG) was constructed under the following assumptions: 1) THA ‘revision’ is dependent on ‘head size’, ‘patient age’, ‘sex’, ‘year of surgery’, surgical ‘approach’, and type of THA ‘fixation’. Choice of ‘head material’ is not expected to affect ‘revision’ due to the short follow-up of the study. 2) Choice of ‘head size’ is dependent on ‘approach’, ‘year of surgery’, ‘sex’, and ‘patient age’ as surgeons operating on older patients through a posterior approach have presumably chosen a larger head in order to, hopefully, reduce the risk of dislocation. Male patients, operated more recently, have probably received a larger head due to their larger acetabulum and because the use of larger heads has become more popular with time. 3) ‘Fixation’ is dependent on ‘year of surgery’ and ‘age’ because patients operated more recently have probably received an uncemented THA, due to the popularization of this technique, and older patients have probably received a cemented THA due to their poorer bone quality. 4) ‘Head material’ is dependent on ‘head size’ and ‘patient age’ because surgeons have probably chosen ceramic over metal heads in younger patients and when choosing larger heads due to the presumed lower polyethylene wear. Provided that our assumptions are correct, adjusting for ‘patient age’, ‘sex’, ‘year of surgery’, and ‘approach’ in the multivariable Cox regression model should block all backdoor pathways (for variables available in our database) confounding the effect of ‘head size’ on ‘revision’.

**Table 2.** Descriptive statistics of study population after propensity score matching. Values number (%) unless otherwise specified

	32-mm head	36-mm head	
	(n = 2,515)	(n = 2,515)	ASDM **^a^**
Follow-up, years **^b^**	2.4 (0.9–4.4)	2.6 (1.1–4.3)	0.03
Mortality	477 (19)	507 (20)	0.03
Age (standard deviation)	70 (11)	71 (11)	0.07
Year of surgery **^b^**	2013	2013	0.05
	(2011–2015)	(2011–2015)	
Female sex	1,570 (62)	1,453 (58)	0.09
Cemented THA	823 (33)	813 (32)	0.02
Cementless THA	1,148 (46)	1,068 (43)	0.06
Hybrid THA	428 (17)	542 (22)	0.1
Reverse hybrid	116 (5)	92 (4)	0.06
MoXLPE **^c^**	2,108 (84)	2,152 (86)	0.06
CoXLPE **^d^**	407 (16)	363 (14)	0.05
Posterior approach	1724 (69)	1705 (68)	0.02

**^a–d^**See Table 1.

**Table 1.** Descriptive statistics of study population before propensity score matching. Values number (%) unless otherwise specified

	32-mm head	36-mm head	
	(n = 8,957)	(n = 3,519)	ASDM ^a^
Follow-up, years ^b^	2.4 (1.0–4.4)	2.3 (1.0–4.0)	0.1
Mortality	1,702 (19)	621 (18)	0.04
Age (standard deviation)	73 (10)	70 (11)	0.2
Year of surgery ^b^	2013	2014	0.1
	(2011–2015)	(2012–2015)	
Female sex	6,266 (70)	1,812 (52)	0.4
Cemented THA	6,276 (70)	813 (23)	0.6
Cementless THA	1,219 (14)	1,729 (50)	0.7
Hybrid THA	430 (5)	885 (25)	0.5
Reverse hybrid	1,032 (12)	92 (3)	0.6
MoXLPE ^c^	7,954 (89)	3,083 (88)	0.04
CoXLPE ^d^	1,003 (11)	436 (12)	0.04
Posterior approach	3,912 (44)	2,599 (74)	0.7

**^a^**Absolute standardized difference in means.

**^b^** Median (interquartile range).

**^c^** Metal head on cross-linked polyethylene.

**^d^** Ceramic head on cross-linked polyethylene.

The primary outcome of our study was the 1st THA revision for any reason and the secondary outcome was the 1st revision due to dislocation. Revision was defined as the exchange or removal of any of the hip prosthetic components. Follow-up time was defined as the time between primary surgery until 1st revision, death, emigration, or December 31, 2016, whichever came first.

### Statistics

Descriptive statistics were performed in SPSS, Version 25 (IBM Corp, Armonk, NY, USA). Mean and standard deviation was used to describe age. Follow-up time was described with median and interquartile range (IQR). PS matching and survival analysis were performed in R software, Version 3.4.4 (R Foundation for Statistical Computing, Vienna, Austria). PS was calculated using the function “matchit” setting head size as the dependent variable and age, sex, year of surgery, type of fixation, type of bearing, and surgical approach as exploratory variables. The calliper was set to 0.15. Patients from the 36-mm group were matched to the 32-mm group using the nearest neighbor method at a 1:1 ratio. Unmatched patients were discarded from both groups. Kaplan–Meier survival curves for the whole observation time were drawn for each head size. The follow-up period was censored at 7 years because after that time point the number of patients at risk in the 36-mm group dropped below 100. After the 7th year of follow-up only 1 revision occurred in the 32-mm group and none in the 36-mm. The Mantel–Cox log rank test was used to compare the survival curves. Univariable Cox proportional hazards models were fitted to calculate the hazard ratio (HR) for head size with 95% confidence intervals (CI) for the period 0–7 years. Multivariable Cox proportional hazards models were also fitted adjusting for patient age, sex, year of surgery, and surgical approach. Despite the minimal differences in these 4 covariates between the head size groups, we chose to put them in the models in order to block all presumed backdoor pathways (for variables available in NARA database) that could confound the effect of head size on THA revision (Figure 4). 32-mm heads were the reference group. Shoenfeld residuals were used to ascertain the proportional hazards assumption. The level of statistical significance was set at alpha = 0.05. There might be some few patients whose revision has not been registered due to lower completeness for revision THA (80–95%) than for primary THA (95–98%) or patients revised in another country than the country of their primary operation. These patients could not be followed up and were considered unrevised.

### Ethics, funding, and potential conflicts of interests

The study was approved by the Regional Ethical Review Board of Gothenburg on October 26, 2016 (reg. ID 858-16). The manuscript was written according to the STROBE (Strengthening the Reporting of Observational studies in Epidemiology) guidelines. No funding specific to this study has been received. 7 co-authors (GT, JNK, GH, OF, KM, ABP, and SO) declare no conflict of interests relevant to this study. 1 co-author (AE) has given paid presentations for and received institutional support from commercial parties (Zimmer-Biomet and DePuy Synthes) related indirectly to the subject of this study. 1 co-author (MM) has given paid presentation for commercial parties (Zimmer-Biomet, Link) related indirectly to the subject of this study.

## Results

**Figure 5.**
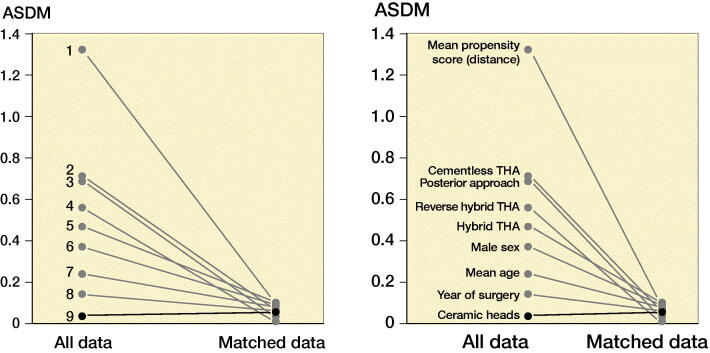
Kaplan–Meier survival function for THA with 32- and 36-mm heads with endpoint revision for any reason (left panel) and revision due to dislocation (right panel).

Up to 7-year follow-up, 119 (4.7%) 1st-time revisions for any reason had occurred in the 32-mm group and 111 (4.4%) 1st-time revisions in the 36-mm group. The Kaplan–Meier survival, although slightly higher for 36-mm heads, did not differ statistically significantly between the 2 head sizes (p_log-rank_ = 0.6). The 7-year survival rate was 92.8% (CI 91.2–94.4) for 32-mm and 93.7% (CI 92.2–95.2) for 36-mm heads (Figure 5, Table 3). Both the univariable and the multivariable Cox regression models (adjusting for age, sex, year of surgery, and surgical approach) showed HR estimates favoring 36-mm heads during the first 7 years after THA, but with CIs extending on both sides of 1 (Table 4).

**Table 4.** Cox proportional hazards models with endpoint revision for any reason and due to dislocation

Outcome Head size	Univariable model HR (CI)	Multivariable model HR (CI)
Revision for any reason		
32-mm	1	1
36-mm	0.9 (0.7–1.2)	0.9 (0.7–1.2)
Revision due to dislocation		
32-mm	1	1
36-mm	0.8 (0.5–1.3)	0.8 (0.5–1.3)

The multivariable model was adjusted for patient age, sex, year of surgery, and type of surgical approach.

HR (CI) = Hazard ratio (95% confidence interval)

**Table 3.** Kaplan–Meier survival estimates (%) at 1, 3, and 7 years for 32- and 36-mm heads with endpoint revision for any reason

Follow-up	Patients at risk	Cumulative revisions	Cumulative survival rate (CI)
32-mm heads			
1-year	1,816	88	95.8 (95.1–96.7)
3-year	1,032	108	94.4 (93.4–95.5)
7-year	223	119	92.8 (91.2–94.4)
36-mm heads			
1-year	1,922	87	95.9 (95.1–96.7)
3-year	1,099	102	95.2 (94.3–96.1)
7-year	140	111	93.7 (92.2–95.2)

CI = 95% confidence intervals.

73 1st-time revisions due to dislocation had occurred during the 1st 7 years of follow-up, of which 61 were done during the 1st year after surgery. 6 were done during the 2nd and 3 during the 3rd year after THA. There were 40 (1.6%) revisions in the 32-mm and 33 (1.3%) in the 36-mm group. With endpoint revision due to dislocation the Kaplan–Meier survival did not differ significantly between the 2 groups (p_log-rank_ = 0.4). The 7-year survival rate for THAs with 32-mm heads was 97.8% (CI 97.0–98.7) and 98.3% (CI 97.6–99.0) for 36-mm heads (Figure 5, Table 5). In the univariable and the multivariable Cox regression model after adjusting for age, sex, year of surgery, and surgical approach, HR estimates were in favor for 36-mm heads, but with CIs including 1 (Table 4).

**Table 5.** Kaplan–Meier survival estimates (%) at 1, 3, and 7 years for 32- and 36-mm heads with endpoint revision due to dislocation

Follow-up	Patients at risk	Cumulative revisions	Cumulative survival rate (CI)
32-mm heads			
1-year	1,816	33	98.4 (97.9–98.9)
3-year	1,032	39	98.1 (97.6–98.7)
7-year	223	40	97.8 (97.0–98.7)
36-mm heads			
1-year	1,922	28	98.7 (98.2–99.2)
3-year	1,099	31	98.6 (98.2–99.1)
7-year	140	33	98.3 (97.6–99.0)

CI = 95% confidence intervals.

## Discussion

In this matched observational study in the NARA database we found that the choice between a 32- or 36-mm head in THA after PFF is not associated with any clinically relevant difference in the risk of revision for either any reason or due to dislocation. The was a trend favoring 36-mm heads, but in absolute numbers this difference corresponded to a decrease in revision rates of only 0.3%. In our opinion, a reduction in revision rates by at least 50% would be clinically relevant, which corresponds to a minimum risk decrease of 2.3% in revisions for any reason and 0.8% in revisions due to dislocations. Besides its small effect size, the difference between 32- and 36-mm heads in our sample is difficult to generalize at a population level due to the lack of statistical significance.

Our study could be insufficiently powered to detect smaller risk differences. PS matching at a 1:1 ratio may have caused a considerable loss of statistical power; however, it was the only matching ratio that allowed an acceptable calliper below 0.2, due to the extent of heterogeneity in the unmatched sample. Moreover, our study has inherent limitations due to the lack of randomization. Unmeasured confounding due to factors unknown to us could have skewed the results. For example, the NARA database does not distinguish among femoral neck fracture, trochanteric, or pathologic PFFs. Most of the trochanteric fractures are treated with internal fixation whereas pathologic fractures might receive a THA. Such cases, although probably very few, might be subjected to increased risk of revision due to poor bone quality. BMI and patient comorbidities that increase the risk of THA revision or dislocation (Peters et al. 2020) are not registered in the NARA database. Surgeons may have used the largest available head size when operating on patients with comorbidities, high BMI, poor compliance, or poor bone quality, hoping to reduce the risk of dislocation. In this case, the accumulation of such patients in the 36-mm group may have disfavored THA survival due to bias by indication. We could not adjust for implant positioning either, since radiographs are not available in our database, but we do not expect the head size groups to differ in terms of prosthesis orientation. 36-mm heads can only be used for cups down to a certain diameter to allow for sufficient thickness of the polyethylene. This could be another source of confounding, provided that the risk for revision varies depending on cup size. Implant size is, however, not recorded in the NARA database and could therefore not be accounted for during PS matching. However, analysis stratified for sex, based on the presumption that females in our study received a higher share of small cups, does not support that this factor had any decisive influence (Table 6, see Supplementary data). A non-posterior approach may include different approaches such as direct anterior, anterolateral, lateral, and transtrochanteric that are not specified in the NARA database. However, no major differences in the risk of THA revision have been reported among these surgical approaches (Berry et al. 2005, Mjaaland et al. 2017, Zijlstra et al. 2017). Patients lost to follow-up due to unregistered THA revisions or having their revision in another country were considered unrevised. These patients are not expected to be overrepresented in any of the head size groups and are therefore not considered a major source of bias. We used revision due to dislocation as an estimate of THA dislocation. However, surgeons might be more reluctant to revise an unstable THA with a larger head, which could favor the survival of 36-mm THA. The median follow-up in our patients (2.5 years) was not long enough to capture time-dependent complications related to head size such as polyethylene wear, osteolysis, and subsequent revision due to aseptic loosening or periprosthetic fracture. However, it was long enough to capture revisions due to early complications such as dislocation, which is the leading cause of 32- and 36-mm THA revision after PFF (Jobory et al. 2019). Finally, heterogeneity in the revision risk related to head size among the 4 national registries could affect the precision of the survival estimates in our sample. We therefore performed Cox regression analyses stratified for each country. There were still no major differences between 32- and 36-mm heads except for Finland, where the risk of revision due to dislocation was found to be lower for 36-mm heads (Table 7, see Supplementary data). This seems to be a result of the lower survival of 32 mm THA in the Finnish Register, compared with the other 3 registries (Figure 6, see Supplementary data). This observation should, however, be viewed cautiously since the Finnish Register contributed with significantly fewer 32-mm THAs than 36-mm (Table 8, see Supplementary data).

Our results confirm 2 previous reports on patients with femoral neck fracture who received THA and where head size could not be identified as a risk factor for revision. In the study from the National Joint Registry (Jameson et al. 2012) head sizes were grouped as < 28, 28, 30 or 32, and ≥ 36 mm. Using 28 mm as reference no association between head size and risk of THA revision for any reason was found. Cementless fixation, on the other hand, was a risk factor for revision (HR 1.8, CI 1.1–3.1). Neither did the study from the Lithuanian register (Cebatorius et al. 2015) find any difference in risk of revision due to dislocation between 28- and 32-mm heads. The use of a posterior approach, however, was associated with 2.7 times (CI 1–5) higher risk of revision due to dislocation. Due to the nature of our study, we cannot make any unbiased estimation of the effect of surgical approach or type of fixation on the risk of THA revision. The absence of further decrease in the revision risk with increased head size, in studies performed exclusively on patients with femoral neck fractures, could be explained by residual confounding or it could also be that any dislocation-preventing effect of using 36-mm heads over 32-mm is so small that it cannot be detected even in larger register studies like ours. Such an effect, if truly present, could probably only marginally address the increased risk of dislocation. Until now, only DMCs that can accommodate considerably larger heads have shown a decreased risk of revision due to dislocation compared with 32- and 36-mm heads in patients with THA after PFF (Jobory et al. 2019). When a higher risk of dislocation is anticipated, DMCs are probably a better option than 36-mm heads but their higher price needs to be considered. Other register studies, performed on either a case mix of hip diagnoses (Hailer et al. 2012, Kostensalo et al. 2013) or exclusively primary osteoarthritis (Zijlstra et al. 2017, Tsikandylakis et al. 2018a), have reported a decreased revision risk due to dislocation with increasing head size, at least up to 32 mm. Our study cannot be compared with such studies as the diagnosis of femoral neck fracture itself is a risk factor for THA revision.

## Conclusion

Choosing a head size of 36 instead of 32 mm does not seem to be associated with any clinically important decrease in the risk of revision due to any reason and not even due to dislocation after THA in patients operated because of PFF. As THA revision due to dislocation is a rare complication, larger studies with better control of confounders, such as register-based randomized control trials, are needed to make sufficiently powered and unbiased estimations of differences in revision risks between head sizes.

## Supplementary Material

Supplemental Material

## Supplementary data

Figures 3 and 6, and Tables 6–8 are available as supplementary data in the online version of this article, http://dx.doi.org/10.1080/17453674.2020.1752559
