# Supplementary material for: Is there a reduction in risk of revision when 36-mm heads instead of 32 mm are used in total hip arthroplasty for patients with proximal femur fractures? A matched analysis of 5,030 patients with a median of 2.5 years’ follow-up between 2006 and 2016 in the Nordic Arthroplasty Register Association
Source: Acta Orthop. 2020 Apr 14;91(4):401–7. doi: 10.1080/17453674.2020.1752559 (PMC8023875; doi:10.1080/17453674.2020.1752559)
Supplement: Supplemental Material [file IORT_A_1752559_SM9939.pdf]

Supplementary data

Table 6. Cox proportional hazards models with endpoint revision for any reason and due to dislocation, stratified by sex

Outcome<br>Head size	Univariable model<br>Females<br>HR (CI)	 Males<br>HR (CI)	Multivariable model<br>Females<br>HR (CI)	 Males<br>HR (CI)
Revision for any reason				
32-mm	1	1	1	1
36-mm	1.1 (0.8–1.5)	0.8 (0.5–1.1)	1.1 (0.8–1.5)	0.8 (0.5–1.2)
Revision due to dislocation				
32-mm	1	1	1	1
36-mm	0.8 (0.5–1.5)	0.8 (0.3–1.8)	0.8 (0.5–1.5)	0.9 (0.4–1.9)

The multivariable model was adjusted for patient age, year of surgery, and type of surgical approach.
HR (CI) = Hazard ratio (95% confidence interval)

Table 7. Univariable Cox proportional model stratified by national register. Hazard ratios refer to 36-mm heads with 32-mm as the reference

Nation	n	Revision for any reason<br>Revisions (%)	 HR (CI)	Revision due to dislocation<br>Revisions (%)	 HR (CI)
Denmark	2,088	128 (6.1)	1.0 (0.7–1.4)	40 (1.9)	1.3 (0.7–2.5)
Norway	712	30 (4.3)	0.4 (0.1–1.2)	4 (0.6)	[a]
Sweden	1,280	28 (2.2)	0.8 (0.4–1.6)	11 (0.9)	0.9 (0.3–2.8)
Finland	950	47 (5.0)	0.8 (0.4–1.4)	18 (1.9)	0.2 (0.1–0.4)

HR (CI) = Hazard ratio (95% confidence interval)
[a] Hazard ratio and CIs could not be calculated.

Table 8. Distribution of head size for each nation contributing to NARA. Values are number (%)

Nation	32-mm THA	36-mm THA
Denmark	1,019 (41)	1,069 (43)
Norway	560 (22)	152 (6)
Sweden	661 (26)	619 (25)
Finland	275 (11)	675 (26)

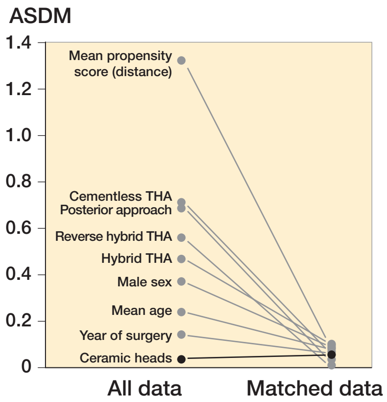

Figure 3. The absolute standardized difference in means (ASDM) between 32- and 36-mm groups before and after matching

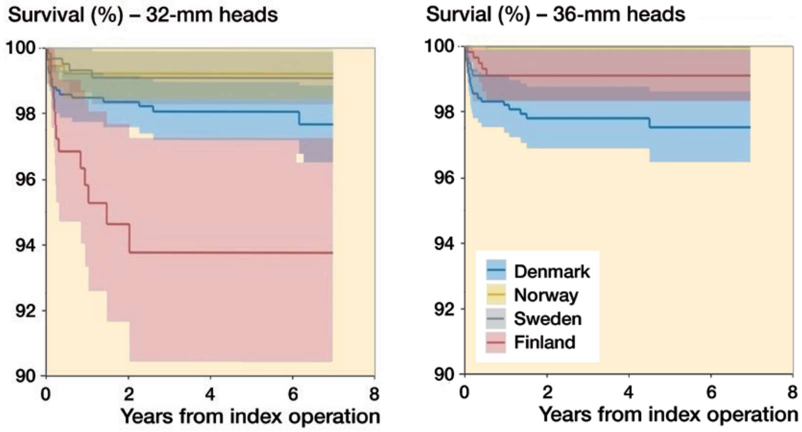

Figure 6. Kaplan–Meier survival for each national register with endpoint revision due to dislocation separately for 32-mm (left panel) and 36-mm (right panel) THA. 36-mm THA seems to perform quite equally among the 4 national registers while 32-mm THA seems to have a poorer Kaplan–Meier survival in the Finish Register compared with the remaining 3 national registers.
